# Nematode Identification Techniques and Recent Advances

**DOI:** 10.3390/plants9101260

**Published:** 2020-09-24

**Authors:** Mesfin Bogale, Anil Baniya, Peter DiGennaro

**Affiliations:** Department of Entomology and Nematology, University of Florida, Gainesville, FL 32611, USA; mazene@ufl.edu (M.B.); anilbaniya1@ufl.edu (A.B.)

**Keywords:** nematode identification, morphology-based methods, DNA-based methods, protein-based methods, image analysis

## Abstract

Nematodes are among the most diverse but least studied organisms. The classic morphology-based identification has proved insufficient to the study of nematode identification and diversity, mainly for lack of sufficient morphological variations among closely related taxa. Different molecular methods have been used to supplement morphology-based methods and/or circumvent these problems with various degrees of success. These methods range from fingerprint to sequence analyses of DNA- and/or protein-based information. Image analyses techniques have also contributed towards this success. In this review, we highlight what each of these methods entail and provide examples where more recent advances of these techniques have been employed in nematode identification. Wherever possible, emphasis has been given to nematodes of agricultural significance. We show that these alternative methods have aided nematode identification and raised our understanding of nematode diversity and phylogeny. We discuss the pros and cons of these methods and conclude that no one method by itself provides all the answers; the choice of method depends on the question at hand, the nature of the samples, and the availability of resources.

## 1. Introduction

Comprising over a million species [1], nematodes are likely the most diverse and numerous metazoans in soil and aquatic sediments. Despite this, nematodes are among the least studied organisms with less than 0.01% of their species diversity described to date [2]. Among some 26,000 described species, about 4100 are plant parasitic, which cause drastic economic losses to all crops [3]. Nematodes are also of significant medical and veterinary importance [4], and free-living nematodes are crucial to nutrient recycling in the environment. Therefore, accurate identification is of paramount significance to understand nematode diversity and design efficient control and management strategies. Traditionally, identification is based on characteristics such as body length, morphology of sexual organs, mouth and tail parts, and other physical characters. This morphology-based classification can prove inadequate due to lack of clear variation among closely related taxa and the need for highly skilled taxonomists, whose number is on the decline [5]. Morphology-based identification is also a demanding endeavor, especially when large numbers of samples are involved. Various sub-organismal (protein- and DNA-based) methods have been employed to supplement or circumvent the limitations associated with morphology-based classification of nematodes. The highly influential work of Blaxter et al. [6] employed sequencing of nematode ribosomal DNA (rDNA) and led to improved understanding of nematode evolutionary relationships and identification. We will not spend time discussing the evolution of nematodes and phylogenetic relationships, but it is important to understand the significance of correct nematode identification and, more to the point, how we define a nematode species. As pointed out by Adams [7] there is a trade-off between an operational species definition and that with a strong philosophical integrity. While there is a justified need to place species within the correct evolutionary lineage, more often, nematode identification techniques are driven by an operational definition of species to assess potential threats to animal and plant health. Here, we review current methods and their progenitors in nematode taxonomic techniques and suggest potential advances.

## 2. Morphological and Image-Based Analyses

### 2.1. Classical Morphological Identification

Classic identification of nematodes is based on morphological and anatomical differences using microscopic image analysis. Morphological identification is among the cheaper identification methods and helps relate morphology with possible function [5]. While most effective for nematodes that have distinct differences, nematodes that share subtle morphological and morphometric differences like body length, presence, and shape of a stylet, the shape of the tail, etc., are difficult to distinguish morphologically. For example, root-knot nematodes (RKN; *Meloidogyne* spp.) were previously diagnosed based on adult female perineal patterns [8,9], i.e., posterior region comprising the vulva-anus area (perineum), tail terminus, phasmids, lateral lines and surrounding cuticular striae; a set of characters that was originally proposed to distinguish among *Meloidogyne incognita*, *M. javanica, M. arenaria* and *M. hapla* [10]. With the discovery of new species, however, perineal patterns became inadequate because perineal patterns (and other morphometric characters; [11]) overlapped between species [12,13]. Currently, RKN species are identified using a combination of morphological and molecular characteristics (e.g., [14,15]).

Another example is in cyst nematodes (*Heterodera* spp. and *Globodera* spp.), which are among the major pathogenic plant parasitic nematodes with worldwide distribution [16]. *Heterodera* and *Globodera* can be distinguished from each other by the morphology of their cysts: lemon shaped in the former and round in the latter [17]. Species identification within *Heterodera* is based on few morphological traits including vulval cone [18], cone top [19], vaginal [20] and lip [21,22] structures. Taxonomic distinction within *Globodera* is mainly based on morphology of cyst and second stage juveniles [23]. Host plant association may also be indicative of the cyst nematode species, though this may be misleading at times as is the case with the cereal cyst nematode group of *Heterodera* [17]. Morphological identification of cyst nematodes requires taxonomic expertise and can be challenging if samples contain mixed species. Moreover, both genera include species complexes whose members are difficult to distinguish based on morphology alone [24,25].

Important morphological identification characters in nematodes include shape of head, number of annules, body length, length of stylet, shape of stylet knob, structure of lateral fields, presence/absence and shape of spermatheca, shape of female tail terminus, shape and length of spicule and gubernaculum [26]. Measurements of these characteristics and processing of samples for this purpose requires skilled taxonomists, whose number is on the decline [5]. Morphology may also be altered due to variation in geographic location, host plant, nutrition, and other environmental factors as is observed among some free-living and plant parasitic nematodes. Concisely, it can be difficult for non-specialists to identify a nematode species with a high level of confidence based on morphology alone [27], and an integration of sub-organismal data such as DNA sequence can be required for accurate identification. However, recent advances in high performance computing may augment human image analyses.

### 2.2. Machine Learning

Advances in machine learning, also referred to as deep learning or artificial intelligence (AI), have opened a new avenue for nematode identification and quantification based on image analysis. The technique is especially suitable for handling large numbers of samples as well as detecting rare and microscopic objects, such as nematode eggs in complex backgrounds.

Machine learning for automated detection of phenotypes takes place in multiple stages. First, a large number of images (of nematodes, their eggs, or cysts) is taken and independently annotated (labeled) by a group of experts to reduce subjectivity. These are then used to build an algorithm that learns (captures) the salient features of the objects from the images in a layer-wise hierarchy while masking (rejecting) the noise in the background. The pattern of interest in the in-put images is then reconstructed using a network model with a supervised learning scheme. Using this technique, Akintayo et al. [28] designed a novel end-to-end Convolutional Selective Autoencoder (CSAE) to identify soybean cyst nematode (SCN) eggs in different backgrounds to cover for variations in background noise across samples from different sources. The authors trained the CSAE to identify SCN eggs using many labeled image segments (patches) that were smaller than the entire image. Information from multiple overlapping local patches was then combined to reconstruct a complete image and determine the existence of an egg in a particular patch. The model correlates pixel intensity values to reconstructed images to show the degree of confidence in predicting the object in the image is indeed an SCN egg. Tests done using two sets of samples collected from regions with different soil properties showed that egg counts done by trained personnel and using this AI technique were comparable at the 95% confidence level.

Another AI technique developed by Hakim et al. [29] using *Caenorhabditis elegans* combines the capabilities of different image processing programs for a fully automated and simultaneous processing of informative phenotypic features in a single platform called WorMachine. The image processor in this platform binarizes, identifies and crops individual worms from still in-put images taken using the bright-field with or without overlapping fluorescent acquisitions. Morphological and fluorescent features are then extracted from the cropped worm masks and analyzed individually by the feature extractor, which also allows labeling of different worms. Based on the features and labels obtained, the machine learner algorithm then conducts a binary classification or scoring of complex phenotypes using principal component analysis (PCA) and *t*-distributed stochastic neighbor embedding (*t*-SNE). The authors distinguished between males (XO), hermaphrodites (XX) and a range of phenotypes in between using fluorescent reporters for sex-specific expression patterns in mutant *C. elegans*. To demonstrate that WorMachine can be used to quantify continuous morphological phenotypes, they used strain CB5362 that is mutated in the sex-determination genes, and quantified intersex phenotypes in worms grown at different temperatures. For each worm, they determined the degree of masculinization from measurements of tail shape, gonad width (larger mid-width in egg-bearing worms), body length and area (males being smaller), brightness of head and tail (darker tails in males in bright-field), analyzed using PCA and *t*-SNE. They reported that the results agreed with those from previous studies, which showed increased masculinity at higher temperatures.

These studies show that AI can play a big role in the detection, quantification as well as classification of nematodes. As such, it will help address some of the limitations associated with the traditional morphology-based classification including the dwindling number of taxonomists, subjective decision making, and provide fast and accurate identification. Ironically, however, generating sufficient training data may present a bottleneck in developing AI due to the declining number of taxonomists. Limitations arising from shared morphological features between taxa would likely remain, but there is the possibility that machine learning will be able to elucidate unique characters discriminating nematodes that have been undetected even by the trained human eye.

### 2.3. Autoflorescence

A potential supplement to traditional light microscopy is the utilization of natural autofluorescence of microorganisms. Bhatta et al. [30] demonstrated that the emission and excitation spectra of the bacterial genera *Lactobacillus* and *Saccharomyces* were distinct. They also reported on the potential of these spectroscopic fingerprints to discriminate between different fungal species within the genus *Saccharomyces* without the need for fluorescent staining. Qazi et al. [31] built on this and demonstrated that eggs of different helminths revealed characteristic florescence when illuminated at different wavelengths ranging from white light to infrared. They also showed that differences in florescence lifetime values (decay in florescence intensity) were diagnostic of the species considered, *Ascaris lumbricoides* and *A. suum*. Qazi et al. [31] concluded that spectroscopic features and lifetime value measurements of autofluorescence in nematodes are promising tools in the taxonomy of these organisms.

## 3. DNA-Based Methods

Many forms of DNA-based methods have been developed for the identification of nematodes (e.g., [32,33,34,35,36,37]). These can be broadly categorized into fingerprint- and nucleotide-based methods. Fingerprint-based methods may include Restriction Fragment Length Polymorphism (RFLP), Amplified Fragment Length Polymorphism (AFLP), Random Amplification of Polymorphic DNA (RAPD) and the use of species-specific primers, which relies on the presence/absence of a PCR amplification product. Except for RFLP, where PCR may not be needed, all fingerprint-based methods involve PCR followed by electrophoresis. The resulting DNA fingerprint, i.e., the pattern of resolution of the DNA fragments, is used for identification and/or phylogenetic analyses of the nematode taxa considered. On the other hand, nucleotide-based methods involve PCR amplification, specific probe hybridizations and sequencing of a region(s) of the DNA, which is then used in phylogenetic analyses. Each of these methods has its own advantages and/or disadvantages compared to other nematode identification methods, DNA-based or otherwise. However, it is notable that nematode sequences have greatly altered our understanding of the evolutionary relationships between taxa [6].

### 3.1. Fingerprint-Based Methods

RFLP analyses can be made using fingerprints generated from genomic DNA (gDNA) digested with one or more endonucleases. Alternatively, fingerprints may be generated from PCR-amplicons (PCR-RFLPs) (e.g., [37,38,39]). gDNA-RFLPs tend to be complex, but potentially reveal more polymorphisms owing to the size of the gDNA template. Also, gDNA-RFLPs do not require knowledge of sequence information a priori, which is not the case with PCR-RFLPs. In both cases, however, care must be taken to let restriction digestions go to completion since incomplete digestions may lead to non-reproducible fingerprints.

The AFLP technique improves upon gDNA-RFLP by selectively amplifying fewer restriction products and producing less-complex fingerprints (e.g., [32,40]). gDNA is digested with two restriction enzymes that produce sticky ends, to which are ligated adaptors. A subset of these adaptor-ligated fragments is then selectively amplified using primer sets that recognize sequences of the adaptors, the sticky ends, and one to three nucleotides inside the restriction sites. As with gDNA-RFLPs, AFLPs do not require prior knowledge of sequence information, and completion of restriction digestions is crucial for reproducible fingerprints.

RAPD involves PCR amplification of gDNA fragments using short (usually 10 bp) primers of arbitrary sequences (e.g., [34,41]). The primers bind to several regions on the DNA, and amplification results if two primers bind on opposite strands of the DNA with their 3′-ends facing each other at a distance that can be traversed by the polymerase. Consequently, fragments of various sizes may be generated, with sizes of the larger fragments dependent on efficiency of the polymerase used. The use of large, intact gDNA template is important for this reason. Because RAPDs are done at lower temperatures, which create lower stringency for primer annealing, reproducibility especially between laboratories also poses a limitation. One advantage of this method is that it does not require prior knowledge of sequence information about the template DNA.

The use of primer sets that amplify a PCR product only in a taxon of interest is commonplace nowadays (e.g., [42,43,44]). Such primer sets can be designed based on fragments that uniquely identify the taxon in fingerprint analyses or based on taxon-dependent nucleotide sequence differences in aligned sequence data. In either case, care must be taken to include as much of the genetic variation within the taxon of interest as well as that of its close phylogenetic relations to ensure specificity of the primer sets. The diagnostic value of species-specific primers is based on amplification of a product only in the species for which they are designed. Therefore, it is necessary to have an internal control for a successful PCR and avoid false negatives by multiplexing the reaction with a second set of primers that amplify a product nonspecifically; after electrophoresis, two bands would be diagnostic of the species of interest while single bands corresponding to the internal control indicate otherwise.

### 3.2. Microarrays and Probe-Based Methods

DNA microarray is a collection of pico-moles of microscopic DNA fragments fixed at defined positions on a solid surface such as a glass slide. For nematode identification, these DNA fragments can be generated from sequence characterized amplified regions (SCARs) and are used as probes to which test samples containing florescent-labeled PCR products or gDNA are made to hybridize in high-throughput diagnostics. Data from hybridized slides are acquired using an array scanner at the emission wavelengths of the florescent dyes used. François et al. [45] investigated the suitability of DNA microarray technique for identification of nematodes using *M. chitwoodi*-specific oligonucleotides as probes. The probes were designed based on nucleotide sequences internal to binding sites of the primer sets used to amplify SCAR and satellite DNA fragments in *M. chitwoodi,* but not in *M. arenaria, M. javanica, M. fallax* and *M. hapla*. In agreement with the specificity of the primer sets in standard PCRs, both SCAR- and satellite DNA-based probes detected *M. chitwoodi* irrespective of the geographical origin of the nematode. However, cross-hybridization with *M. chitwoodi* targets was observed when satellite DNA-based probes designed from the pMfFd satellite DNA family of *M. fallax,* a closely related species, was used. This shows that careful selection of probes is important. This is the only study that we came across where DNA microarray technology was used in nematode diagnostics.

TaqMan qPCR also employs labelled DNA probe(s) for the detection and quantification of nematodes. At the start of TaqMan qPCR, the labelled probe binds to the template DNA within the site circumscribed by the primers. As the reaction progresses and the polymerase reaches the probe, its endogenous 5′ nuclease activity cleaves the probe, separating the dye from the quencher at the 3′-end of the probe. With each PCR cycle, more dye molecules are released, resulting in an increase in fluorescence intensity proportional to the amount of amplicon synthesized. The inclusion of probe(s) makes the technique more specific than standard PCRs and the amount of florescence detected can be used to quantify the number of nematodes in the sample. Primers and probes may be designed from aligned sequence data (e.g., [46]) as described above for species-specific primers, or from SCARs (e.g., [47]). Using this technique, Sapkota et al. [46], for example, developed a real-time PCR assay for the detection of M. hapla in soil and in root galls. They were able to differentiate *M. hapla* DNA from among those of 14 other *Meloidogyne* spp. included in their study except for *M. minor*. Based on aligned sequences from the 14 species, the authors concluded that the *M. minor* DNA must have been contaminated with that of *M. hapla* for amplification to result using these primer sets and probe. The authors reported *M. hapla* DNA extracted from 250 mg of soil (containing the equivalent of a third of an egg) could be detected by this technique. Similar studies have been carried out for other nematode taxa as well, which reported on the suitability of TaqMan qPCR for detection and quantification of nematode taxa (e.g., [47,48]).

### 3.3. Sequence-Based Methods

Sequence-based methods may involve analyses of nucleotide sequence information from specific segment(s) of the nuclear DNA, mitochondrial DNA (mtDNA), or the whole genome (for examples of gene regions and the corresponding primer sets, see: [42,49,50,51,52,53,54]). The rDNA and mitochondrial cytochrome c oxidase subunit I (*COX1*) genes are preferred by most studies (e.g., [54,55,56,57,58]) for diagnostic purposes because they have variable regions circumscribed by conserved ones. The higher level of sequence diversity in the variable region makes *COX1* preferable for resolution at lower taxonomic levels such as species and subspecies groups (e.g., [59]), while the higher level of sequence conservation in the flanking regions, which allows for ‘universal’ primers to be designed [56], has made the rDNA more suitable for use in wider taxonomic levels. The bulk of the sequence variability in the rDNA is harbored in the internal transcribed spacer (ITS), which is interrupted by the 5.8S coding region in the rDNA cistron into ITS1 and ITS2 [60], making the ITS useful in molecular systematics of closely related nematode species (e.g., [61,62,63]). ITS2 alone has been used for species diagnosis in *Caenorhabditis* [64] involving genetic crosses of newly collected isolates with known biological species, though the authors do not advocate for the use of ITS2 as an absolute criterion for species diagnosis because of the potential that distinct species may share identical ITS2 sequences. An added advantage of *COX1* and rDNA is that both genes occur in multiple copies in nematode genomes enabling PCR amplifications form small amounts of DNA templates such as that can be obtained from single nematodes. Sequence information generated is then used in character-based or phylogenetic analyses to resolve and/or identify the taxa involved; the latter analysis allows for evolutionary inferences.

The rDNA encompasses conserved coding regions (28S, 18S, and 5.8S subunits) and variable non-coding regions (ITS and ETS; the external-transcribed region) organized as tandem repeats, with intergenic spacers separating the repeating units [60]. As mentioned above, the rDNA provides phylogenetic resolution at a wide range of taxonomic levels and allows ‘universal’ primers to be designed for use in these taxa. This has led researchers to propose different regions of the rDNA for use as DNA barcode in different organisms; unique nucleotide sequences that can potentially be used to identify each species. Proposed DNA regions include ITS for fungi [65], 16S for bacteria [66], and 18S for nematodes [67,68]. The barcode region used in animals is the *COX1* region [69]. As such, DNA barcodes use sequence information from defined regions of the DNA to identify species using primers that are applicable for the broadest possible taxonomic group. Intraspecific variations should be smaller than interspecific variations in the barcode region.

Floyd et al. [67] used sequence information from the 18S (small subunit; SSU) to group soil nematodes into molecular operational taxonomic units (MOTUs). Each of these MOTUs was comprised of a cluster of sequences that differed from one another by less than three bases over aligned sequence data. The aligned data contained 349 to 396 nucleotides after removal of gaps, ambiguous characters and unresolved base calls from 450–500 nucleotide-long raw sequences generated using primer SSU94 [6]. MOTU content was predicted from neighbor joining trees generated using absolute character differences as a measure of distance. The authors reported that MOTUs largely corresponded with morphologically defined species or genera. Powers et al. [70] also studied a region of the 18S as a potential barcode for nematodes of suborder *Criconematina*. This region does not overlap with that used by Floyd et al. [67] and lies closer to ITS1. The authors used both phylogenetic and character state differences to define MOTUs. Among the 132 polymorphic sites in the aligned dataset, 56 were singletons and defined 56 MOTUs, each consisting of identical sequences. Most clades did not have strong statistical support, and morphologically identified species did not correspond with phylogenetically supported clades except for Clade B. Apart from a single MOTU, Clade B exclusively consisted of *Discocriconemella limitanea*, represented by 11 MOTUs, which may be cryptic species according to the authors. Conversely, some individual MOTUs identified a complex of species. For example, MOTU 76 corresponded to *Ogma* spp. that have scales arranged singularly in longitudinal rows along the length of the body, or arranged in rows consisting of clusters of 4–6 scales, or with scales densely packed on the annules forming a continuous elongated fringe.

The value of a barcode is directly related to the taxonomic rank it can effectively be applied to. The regions of SSU tested for their potential as barcodes by Powers et al. [70] and Floyd et al. [67] resolved the respective soil nematodes into named taxa and/or MOTUs. However, it is evident that MOTUs cannot be compared between the two studies because they were established based on incongruent sequence information; a phylum-wide barcode would be more powerful, but possible only if taxa representing the whole phylum were analyzed for the same DNA region. The sequence heterogeneity in individual nematodes that was reported by Powers et al. [70] is also suggestive of sequence variation among different copies of the SSU in the rDNA tandem repeat. Though, Dorris et al. [71] and Floyd et al. [67] stated that there is no evidence in nematodes of one species carrying more than one very distinct sequence variant. Bik et al. [72], however, have demonstrated that there exists intragenomic rRNA polymorphism and copy number variation in nematodes, and that the existence of minor variant gene copies in the rRNA repeats presents substantial challenges for biodiversity estimates and the analysis of marker-based datasets. Care must be taken to exclude such variable sites during analyses if the variation is greater than the cut-off value (see below). Another issue that needs to be addressed is how to interpret the barcode. DeSalle et al. [73] contend that a non-tree-based population aggregation analysis (PAA; [74]) is the most appropriate approach because tree-building approaches are flawed for many reasons. Firstly, morphology-based methods are character-based rendering the union of classical methods and distance-based DNA barcoding difficult. Secondly, tree-building methods are hierarchical while the underlying system consisting of individuals and populations is not. Thirdly, cut-off values are rather subjective; there is no objective set of criteria to delineate taxa when using distances. DeSalle et al. [73] emphasize that the best approach is to look for diagnostic characters in the aligned sequences themselves.

A great advantage of sequence-based methods is that sequence information is stored in publicly available databases such as GenBank (ncbi.nlm.nih.gov) and NEMBASE (nematodes.org). This facilitates identification of nematodes based on sequence information through comparison with that available in these databases. Accuracy of identification, however, depends on the quality of sequences deposited in the databases and the authenticity of the taxa the sequences originated from.

Most journals require that sequences be submitted to open-access databases as part of the publication process. But there is no such requirement for alignments. Unavailability in these databases of aligned sequence datasets may affect identification, especially that based on character states. This is because though alignments are generated using software, they invariably need manual editing particularly when larger datasets containing ambiguous sites are involved, which may introduce variations in alignments.

While gene-specific sequence information is commonly employed at lower taxonomic levels, there is a growing effort to include whole mitochondrial or whole genome sequence information at all taxonomic levels now that sequencing has become more affordable. Comparative genomics enables retrieval of additional information such as synteny and gene order for the investigation of underlying evolutionary mechanisms like inversion, translocation, fusion, and so on, in addition to aiding a more advanced understanding of nematode biology. After the call to sequence 959 nematode genomes by Kumar et al. [75], progressively larger number of nematode genomes have been sequenced. It would be advantageous if whole genome sequencing projects involve morphological type specimens where possible as the availability of sequence information from type specimens in the databases would help improve the accuracy of sequence-based identification of nematode samples.

## 4. Protein-Based Methods

Like DNA-based methods, protein sequences, mass-to-charge ratios, and immunological techniques focus on using unique protein composition and structures to delineate nematode species. Proteins provide a reduced vocabulary compared to DNA due to redundancy of the genetic code; however, the alphabet used is vastly more complex, utilizing 20 plus characters compared to the four DNA bases. Additionally, protein structure and post-translational modifications increase the potential diversity available to define nematode species and facilitate identification. Nonetheless, the requisite specialization in protein-based techniques is often a significant deterrent.

### 4.1. Isozyme Analyses

Enzyme phenotypes were among the first non-morphology-based methods used for the identification of nematodes. Briefly, this technique involves the extraction of soluble proteins from whole nematodes in buffer solutions, resolving the resulting extracts by starch or polyacrylamide gel electrophoresis followed by staining for specific enzymes. This electrophoretic method, also known as Multi-locus Enzyme Electrophoresis (MEE), relies on the migration patterns of isozymes, owing to differences in electrical charge, molecular weight, and conformation stemming from slight variations in amino acid compositions. The most commonly utilized enzymes were esterases [76], though malate dehydorgenase, superoxide dismutase, and glutamate-oxaloacetate transaminase have also been employed to various degrees [76,77]. This technique supplemented morphological methods and shed light in the phylogenetic relationships, especially among the major species in the genus *Meloidogyne*. However, the method was still cumbersome and time consuming; and the need to include known samples for reference purposes are among its limitations [76].

### 4.2. Two-Dimensional Gel Analyses

Two-dimensional gel electrophoresis (2-DGE) has been employed in taxonomic studies of nematodes. The technique allows resolution of complex protein mixtures by charge using isoelectric focusing in one-dimension followed by mass-based resolution in a dimension perpendicular to the first. The resolution pattern is then compared among isolates to determine similarities/differences, which can be scored as presence/absence for phenetic and/or cladistic analyses of the resulting data matrix. Navas et al. [78] used 2-DGE to show proteomic variations among 18 root-knot nematodes representing four species. They demonstrated that some of these variations were species-specific, while other variations revealed evolutionary relationships among the different species.

The technique has a number of pros and cons as applied to nematode taxonomy. One of the pros of 2-DGE is that it allows evolutionary inferences to be made about the taxa considered. Species-specific polypeptides can also be excised and analyzed using mass spectrometry (see below) allowing inferences to be made about the encoding genes. The cons include that the number of polypeptides resolved, and the polymorphism observed depend on the procedure used and the number of samples analyzed. For example, the number of polypeptides Navas et al. [78] observed among the 18 isolates ranged from 73–203. The authors stated that scoring the spots was difficult at times because it was hard to assess if some of the observed differences were real or due to deformations in the gel. For this reason, they scored only 95 spots that were consistently expressed in the two replicates they used for each nematode. Thirty-seven of these spots were monomorphic and thus uninformative. Considering that two of the species in their study were represented by single isolates only, it can be concluded that both the total number as well as the number of informative spots would have been different from what they observed had they used larger number of isolates. 

### 4.3. Mass Spectral Analyses

Matrix-assisted laser desorption/ionization (MALDI) is an ionization technique, which uses laser energy-absorbing matrix to generate gaseous ions from large molecules in solid state. Embedded in a suitable matrix, the sample is applied onto a plate and irradiated with pulsed laser resulting in vaporization of the sample and the matrix material. Molecules are ionized by loss/gain of proton(s) in the hot plume of ablated gases and accelerated into a mass spectrometer for detection. Time of flight mass spectrometer (ToF-MS) measures the time taken by these ions to reach the detector as determined by the mass/charge (*m*/*z*) values, with smaller and/or more charged ions travelling faster. Since MALDI results in minimum fragmentation, the ions generated are predominantly non-fragmented and single-charged, which makes it easy to determine parental ion masses from mass spectra [79].

The basis of taxonomic identification using MALDI-ToF-MS is the ability to detect protein/peptide ions or protein profiles that are diagnostic to the taxa being considered. Perera et al. [80] used intact second stage juveniles (J2s) and/or proteins extracted from these using various organic solvents and discriminated between *Anguina tritici, A. funesta* and *M. javanica* based on unique peaks in their spectra and/or the spectral profiles. However, the authors advised that care must be taken when selecting the solvent for protein extraction and the matrix material for the MALDI as reproducibility and quality of the spectra vary with the material used. Ahmad et al. [81] built on this study to show that single *M. incognita* nematodes (an adult female or a J2) washed or unwashed, crushed or intact, can be used for diagnostics using MALDI-ToF-MS. Their study revealed that protein profiles differed between adults and J2s, each with its own diagnostic peaks; more masses and stronger peaks were also observed when washed and/or crushed samples were used. Both studies reported that careful optimization of instrument settings is also crucial.

Navas et al. [78] generated MALDI-ToF-MS spectral profiles for species-specific proteins obtained from excised 2-DGE gels to identify the proteins for use as biomarker molecules. Their attempt to identify the proteins using similarity matches, however, returned no hit for lack of sufficient information in the databases at the time. A similar study involving 2-DGE and MALDI-ToF-MS analyses of proteomes of two nematomorph species, *Paragordius tricuspidatus* and *Spinochordodes tellinii,* was carried out by Biron et al. [82]. Biron et al. [82] reported that while 36.2% of total protein spots on the 2-DGE analyses were shared between the two hairworm species, 38.0% were specific to *P. tricuspidatus* and 25.8% to *S. tellinii*; a genetic distance of 0.47 separated the two species confirming the distant relationship reported previously for these species. Unlike Navas et al. [78], Biron et al. [82] were successful in identifying MS fingerprints of proteins obtained from excised gel plugs using similarity searches in the databases.

These studies [78,80,81,82] have demonstrated that 2-DGE coupled with MALDI-ToF-MS provide a powerful tool in nematode taxonomy. The methods allow for inferences to be made regarding evolutionary relationships among taxa as well as for development of species-specific markers. Results, however, can be affected by a number of factors including the protein extraction method, the quality of 2-DGE runs, and setup of instrument. Protein expression profiles are also known to differ depending on the developmental stage of nematodes and the growth conditions. 

### 4.4. Serological Analyses

Since Bird [83] first reported on the possibility of generating antisera against nematodes, the application of poly- and monoclonal antibodies (mAbs) has been explored by several researchers with mixed results (for a summary see [84]). For example, Lee [85] reported that antiserum raised against *M. incognita* did not produce the trademark arc-shaped precipitation band when paired with antigens from another species within the same genera, *M. hapla,* in the Ouchterlony double diffusion assay, indicating a lack of cross-reactivity. However, it was noted that the apparent specificity may be due to the small number of nematodes used in the assay. Further studies [86,87,88] also confirmed a lack of specificity in reactivity of antisera from *Meloidogyne* spp. Similar mixed results were also observed among cyst nematode *Heterodera* and *Globodera* species (summarized in [84]). Cross-reactivity of polyclonal antisera raised against whole macerated nematodes, including the associated microbiome and metabolites, in their bodies is to be expected.

The development of the hybridoma technique by Kohler and Milstein [89] raised the hope of the nematology community to develop mAbs for diagnostic purposes. The technique involved isolating mature B-cells from animals immunized with nematode antigens, fusing these B-cells with mouse lymphoid tumor cells to produce hybridomas that can be maintained indefinitely in vitro for continuous production of the antibodies. mAbs provide more specificity depending on the immunogen the antibodies were raised against. mAbs were raised against a variety of agriculturally important nematodes including *H. glycines* [90], *M. incognita* [91], *G. rostochiensis* and *G. pallida* [84] using the hybridoma technique. Schots et al. [84] reported that some mAbs differentiated between *G. rostochiensis* and *G. pallida* isolates. The authors also showed that these mAbs were so sensitive that protein equivalents of less than one egg were detected using immunoassays. The hybridoma technique becomes cumbersome with increasing number of nematode samples. The low proportion of successful fusions obtained between B- and tumor cells also presents a handicap. Next-generation sequencing technologies may prove to revitalize this line of nematode identification techniques as single B-cell receptor sequencing (scBCR-seq) can reconstruct antigen binding site sequences for comparative studies [92].

## 5. Conclusions

The purpose of taxonomy is to understand biodiversity, categorize organisms, and aid the communication of biological information. Scientific naming is a prerequisite for communication in taxonomy, and valid naming is only possible with type specimens and corresponding morphological information. However, this is not always possible, particularly when dealing with environmental samples (eDNA). Furthermore, it is now generally accepted that there are insufficient morphological features to describe biological diversity, and the use of molecular information to supplement and/or circumvent this limitation is commonplace. Nonetheless, a taxon is more meaningful if its members possess unique biological features, rather than the taxon only representing a group of individuals sharing similar morphological or molecular features.

Morphology-based classification forms the foundation of taxonomy. It has benefited from recent advances in image analysis. AI helps circumvent limitations associated with the scarcity of highly qualified taxonomists and enables objective decision making, coupled with fast and accurate identification. Spectroscopic features and lifetime value measurements of autofluorescence also provide additional traits that can be exploited for identification purposes. 

The relative ease of molecular methods (Table 1) has led to the recognition of many new taxa; some, based on sequence information alone. These taxa would have been impossible to describe morphologically not only for lack of taxonomists and sufficient morphological differences, but also because members of these taxa are difficult to culture. Taxa identified using different molecular approaches, however, are not always congruent; for example, when sequence information from different regions of the DNA is used in different studies, or when sequence data generated from the same DNA region are analyzed differently between studies. Likewise, taxa based on morphological features do not always correspond with those based on molecular information and vice versa. Consequently, no single method by itself provides all the answers all the time; and the choice of method(s) depends on the question asked, the nature of the samples and the availability of resources.

If the question at hand is identification of a nematode sample, the most direct approach would be to examine the sample microscopically and assign the nematode to the lowest taxonomic rank possible. The source of the sample may also provide a clue in this regard. However, this may require some level of taxonomic expertise. Based on this information, a molecular technique may then be employed to identity the nematode to species or even subspecies level. If the question has to do with quarantine, molecular methods that are specific to the quarantined nematode species may be employed to ascertain whether the nematode at hand is quarantined. If the objective is assessment of diversity in a field population(s), any of the fingerprinting techniques and/or sequence analyses based on one or a few genes may do. High-throughput sequencing using second or third generation technologies and the appropriate bioinformatic techniques are useful to study the diversity of nematodes in an environmental sample (eDNA).

## Figures and Tables

**Table 1:** Comparison of different nematode identification methods.

Method	Expertise	Cost	Resolution
Morphological and Image-Based			
Classical Morphometrics	High	Low	Medium
Machine Learning	High	Low	Medium
Autoflorescence	High	Low	Medium
DNA-Based			
Fingerprint	Medium	Medium	Medium
Microarray / Probe-Based	Medium	Low	Medium
Sequencing	Medium	High	High
Protein-Based			
Isozyme Analyses	Medium	Medium	Medium
2-D Gel Analyses	Medium	Low	Medium
Mass Spectrometry	Medium	Medium	Medium
Serological Analyses	High	High	Medium
